# Comparison of Conventional Statistical Methods with Machine Learning in Medicine: Diagnosis, Drug Development, and Treatment

**DOI:** 10.3390/medicina56090455

**Published:** 2020-09-08

**Authors:** Hema Sekhar Reddy Rajula, Giuseppe Verlato, Mirko Manchia, Nadia Antonucci, Vassilios Fanos

**Affiliations:** 1 Neonatal Intensive Care Unit, Department of Surgical Sciences, AOU and University of Cagliari, 09042 Cagliari, Italy; hemasekhar09@gmail.com (H.S.R.R.); vafanos@tin.it (V.F.); 2 Marie Sklodowska-Curie CAPICE Project, Department of Surgical Sciences, University of Cagliari, 09042 Cagliari, Italy; 3 Unit of Epidemiology and Medical Statistics, Department of Diagnostics and Public Health, University of Verona, 37129 Verona, Italy; giuseppe.verlato@univr.it (G.V.); nadia.antonucci@univr.it (N.A.); 4 Section of Psychiatry, Department of Medical Science and Public Health, University of Cagliari, 09125 Cagliari, Italy; 5 Department of Pharmacology, Dalhousie University, Halifax 6299, NS B3H 4R2, Canada

**Keywords:** machine learning, medicine, healthcare, diagnosis, drug development, personalized treatment, autonomous technology

## Abstract

Futurists have anticipated that novel autonomous technologies, embedded with machine learning (ML), will substantially influence healthcare. ML is focused on making predictions as accurate as possible, while traditional statistical models are aimed at inferring relationships between variables. The benefits of ML comprise flexibility and scalability compared with conventional statistical approaches, which makes it deployable for several tasks, such as diagnosis and classification, and survival predictions. However, much of ML-based analysis remains scattered, lacking a cohesive structure. There is a need to evaluate and compare the performance of well-developed conventional statistical methods and ML on patient outcomes, such as survival, response to treatment, and patient-reported outcomes (PROs). In this article, we compare the usefulness and limitations of traditional statistical methods and ML, when applied to the medical field. Traditional statistical methods seem to be more useful when the number of cases largely exceeds the number of variables under study and a priori knowledge on the topic under study is substantial such as in public health. ML could be more suited in highly innovative fields with a huge bulk of data, such as omics, radiodiagnostics, drug development, and personalized treatment. Integration of the two approaches should be preferred over a unidirectional choice of either approach.

## 1. Introduction

**Figure 1:**
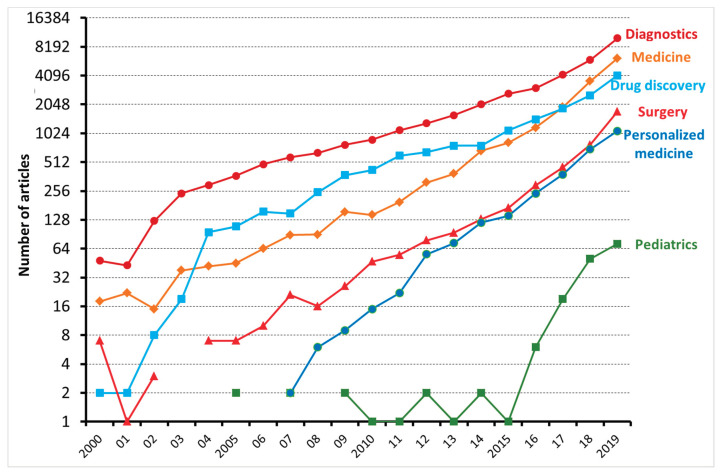
Number of articles, reviews, and editorials, dealing with machine learning and either diagnostics, medicine, drug discovery, surgery, personalized medicine, and pediatrics, published between 2000 and 2019 and indexed on the Web of Science.

Machine learning (ML) is a type of artificial intelligence (AI) consisting of algorithmic approaches that enable machines to solve problems deprived of explicit computer programming [1]. ML is becoming increasingly relevant in medicine as it can optimize the trajectory of clinical care of patients affected by chronic diseases and might inform precision medicine approaches and facilitate clinical trials. As shown in Figure 1, the number of articles applying ML to the medical field has been exponentially increasing, especially with regard to diagnostics and drug discovery. According to Accenture data, vital medical health AI applications can possibly create USD 150 billion in yearly savings for the United States healthcare sector by 2026 [2]. These data show that the healthcare industry can heavily leverage the possibilities provided by ML. This might also explain why AI companies are being increasingly involved in the area of medicine, from diagnosis to treatment and drug development. For instance, convolutional neural networks (used in image recognition and processing) have been able to effectively improve the diagnostic process of diabetic retinopathy [3,4]. Another example is rehabilitation, where learning agents can be trained to run by controlling the muscles attached to the virtual skeleton. Ideally, doctors might predict if a patient is able to walk, jump, or run properly after a specific treatment. Furthermore, data obtained during phases of rehabilitation might be later used to project new, AI designed, leg prostheses.

AI uses multiple layers of non-linear processing units to “teach” itself how to understand data, classify the records, or make predictions [5]. Thus, AI can produce electronic health records (EHRs) data and unstructured facts to make predictions about a patient’s health. For instance, AI can rapidly read a retinal image or flag cases for follow up when several manual reviews would be too cumbersome [6].

When applied to big data, AI offers the promise of unlocking novel insights and accelerating breakthroughs. Paradoxically, although an unprecedented quantity of data is becoming available, only a fraction is being properly integrated, understood, and analyzed. The challenge lies in harnessing high volumes of data, integrating them from hundreds of sources, and understanding their various formats. AI offers potential for addressing these challenges, since cognitive answers are explicitly intended to integrate and analyze big datasets. AI can understand diverse types of data such as lab calculations in a structured database or the script of a scientific publication. These software solutions are trained to understand technical, industry-specific content and use advanced reasoning, predictive modelling, and ML techniques to advance research.

Indeed, AI can be applied to big data using different approaches. When it comes to the effectiveness of ML, the rule of thumb is that the more data, the more accurate the prediction. Although this is an oversimplification, it is evident that the healthcare sector is sitting on a data goldmine. Estimates are that big data and ML in pharma and medicine could generate a value of up to USD 70 billion to 100 billion annually [7], given the downstream effects of these approaches.

One main difference between ML and traditional statistical methods lies in their purpose, as the former remains focused on making predictions as accurate as possible, while the latter are aimed at inferring relationships between variables [8].

However, the key difference between traditional statistical approaches and ML is that in the latter, a model learns from examples rather than being programmed with rules. For a given assignment, samples are provided in the form of inputs (called features) and outputs (called labels). For instance, digitized slides read by pathologists are rehabilitated to features (pixels of the slides) and labels (e.g., data indicating that a slide comprises evidence of deviations indicating cancer) [9]. Using algorithms for learning from observations, computers then govern how to accomplish the mapping from features to labels in order to create a model that will generalize the data, such that an assignment can be achieved properly with new, never seen before inputs (e.g., pathology slides that have not yet been read by a human). This process is called supervised machine learning.

When predictive accuracy is critically significant, the ability of a model to find statistical patterns through millions of features and instances is what enables superhuman performance. Nonetheless, these patterns do not necessarily relate to the identification of underlying biologic pathways or modifiable risk factors that might facilitate the development of new therapies [9].

A crucial difference between human learning and ML is that humans can learn to make general and complex associations from small amounts of data. Machines, in general, require several more samples than humans to acquire the same task, and machines are not capable of common sense. The flipside, however, is that the machine can learn from massive amounts of data: it is perfectly feasible for an ML model to be trained with the use of tens of millions of patient charts warehoused in EHRs, with hundreds of billions of data points, deprived of any lapses of attention, while it is very challenging for a human physician to understand more than a few tens of thousands of patients in a complete career. The performance of well-developed conventional statistical approaches needs to be evaluated and compared with ML in terms of predictivity of clinically relevant outcomes (e.g., survival, response to treatment, patient-reported outcomes (PROs), etc.).

In this narrative review, we aim to offer an expert perspective on the comparison of traditional statistical methods with ML, and their corresponding advantages and limitations in medicine, with a specific focus on the integration between the two approaches and its application to illness detection, drug development, and treatment. To this end, we have selectively reviewed the literature on this topic, presenting evidence illustrating the difference between traditional statistical methods and ML in healthcare.

## 2. Advantages of Traditional Statistical Methods over ML

Traditional statistical approaches have the advantage of being simple to understand. Indeed, they usually take into account a small number of clinically important variables and they produce “clinician-friendly” measures of association, such as odds ratios in the logistic regression model or the hazard ratios in the Cox regression model. Traditional statistical approaches allow us to easily understand the underlying biological mechanisms.

On the other hand, the results of ML are often difficult to interpret. Lack of interpretability is particularly evident in neural networks, but it is less pronounced in least absolute shrinkage and selection operator (Lasso) regression. Moreover, computation to find the minimum of the cost function of neural networks is quite complex and time-consuming, depending on the type of cost function chosen, the number of nodes and layers of the neural network, and the number of training observations [10]. Furthermore, ML algorithms entail data pre-processing, training on datasets, require large datasets, and iterative refinement with regard to the real medical problem [1]. ML techniques can also lead to overfitting, i.e., to the production of a model too closely related to the underlying dataset. This phenomenon can limit the possibility of generalizing the model to different datasets, and hence, making predictions [11]. An appropriate balance between the training set and the validation set is necessary to avoid this problem.

## 3. Advantages of ML over Traditional Statistical Techniques

ML techniques have large flexibility and are free from a priori assumptions, while traditional statistical methods rely on strong assumptions, such as the type of error distribution, additivity of the parameters within the linear predictor, and proportional hazards. These assumptions are often not met in clinical practice and they are often overlooked in the scientific literature. For instance, the assumption of proportional hazards has been violated when studying survival in gastric cancer patients, as the prognostic significance of the depth of tumor invasion and nodal status tends to decrease with increasing follow-up, while the histology and the loss of TP53 gene acquire prognostic importance after at least two years of follow-up [12].

ML has the advantage of taking into account all the available information on a particular field. Traditional statistical approaches, even those at the top of the pyramid of evidence, often fail because they make a priori selection of the variables to be considered. For instance, a Cochrane review, dealing with the extension of lymphadenectomy in gastric cancer surgery, was criticized and later withdrawn mainly because it failed to take into account the quality of surgical procedures under comparison [13]. ML is particularly suited when there are few observations and many predictors, such as in genomics, transcriptomics, proteomics, and metabolomics [14]. In such a situation, traditional regression models show several limitations, especially for the choice of the most important risk factors. Therefore, in building ML predictive models, it is possible to use numerous approaches to apply also on small datasets.

ML can also easily address interactions, which are difficult to investigate with traditional statistical methods that can mostly address interactions between the main determinant and single potential confounders. For instance, the effect of the surgical approach on survival in gastric cancer patients is modulated by tumor stage and histology [15]. However, this second-order interaction is difficult to highlight within a Cox model [16], as the interaction between lymphadenectomy and histology becomes apparent after the first two years of follow-up.

Furthermore, ML algorithms have the ability to analyze various data types (for instance, imaging data, demographic data, and laboratory findings) and integrate them into predictions for illness risk, diagnosis, prognosis, and applicable treatments [1].

## 4. Different Indications for the Two Computational Approaches

Taking into account the strengths and limitations discussed above, different fields of application can be proposed for traditional statistical techniques and ML. Traditional statistical approaches could be more suitable than ML when: (1) there is substantial a priori knowledge on the topic under study; (2) the set of input variables is limited and rather defined in the current literature; (3) the number of observations largely exceeds the number of input variables. This situation is typically encountered in public health research, especially when performed on large healthcare utilization databases [17,18]. 

On the other hand, ML techniques have proven to be more appropriate in “omics” [19], where numerous variables are involved (genes, RNA molecules, proteins, metabolites). Indeed, with a large number of interactions (such as polygenicity and epistatic effects in genomics), ML might help disentangle the complex relationships between these components in determining their effect on the main outcome (i.e., the illness risk).

Traditional statistical approaches are appropriate when the set of predictors tends to be defined a priori on the basis of available reliable evidence on the specific topic. For instance, most articles dealing with gastric cancer surgery include a fixed set of covariates in survival models, comprising sex, age, tumor site, histology, and stage [12]. The selection of variables is important to avoid the introduction of strongly collinear variables, such as tumor stage and surgical efficacy (completeness of tumor removal), and this is usually done on the basis of a priori knowledge, as techniques to compare non-nested models, such as Akaike Information Criterion, are rather limited. This approach makes the studies more comparable: for instance, the use of the same prognostic factors allows the comparison of datasets collected in different countries and makes easy to develop internationally accepted prognostic scores [20]. On the other hand, this approach could slow down the progress of clinical research, as few novel prognostic factors are addressed by each research project.

ML allows us to take into account a huge bulk of potential predictors, avoiding an a priori choice among them. Hence, ML is more suited for big steps in diagnostics and therapeutics. ML has given an important contribution to the rapidly progressing therapeutic revolution fostered by “omics”. However, whatever boundaries we can establish today between traditional statistics and ML, these will be surely overcome in the next future.

## 5. Integration between the Two Approaches

A traditional statistical approach requires us to choose a model that incorporates our knowledge of the system, and ML requires us to choose a predictive algorithm by relying on its empirical capabilities [19]. Justification for an inference model generally rests on whether it sufficiently captures the characteristics of the system. The choice of algorithm in pattern learning frequently hangs on measures of previous performance in similar scenarios. Inference and ML are complementary in pointing us to biologically meaningful conclusions.

Of note, traditional statistical approaches and ML are often used in sequence. When trying to differentiate groups of patients based on their proteomic or metabolomics profile, classical statistical techniques are first used for preliminary screening, while ML is used to finalize the analysis.

For instance, Fabris et al. have recently identified a set of urinary proteins that allow the discrimination between two different renal diseases, nephrolithiasis and Medullary Sponge Kidney [14]. Remarkably, this result was achieved on a very small series (22 patients with MSK and 22 patients with idiopathic calcium nephrolithiasis), analyzing a huge bulk of urinary proteins (*n* = 1529). Traditional statistical techniques (multidimensional scaling, volcano plot, and ROC curves) allowed them to reduce the set of urinary proteins considered from 1529 to 16, while Support Vector Machine (SVM) permitted a further reduction to 5 proteins. In a subsequent study on the same topic, Bruschi et al. first used partial least squares discriminant analysis and then SVM [21].

## 6. Applications of ML in Medicine

### 6.1. Diagnostic Process

The ability of ML to detect diagnostic models reaching the level of clinical accuracy remains an objective not yet achieved, but seemingly feasible. This objective faces the challenge of finding ways to work with all the available data. This highlights the relevance of interdisciplinary collaborative work. In the area of brain diseases like depression, the Predicting Response to Depression Treatment (PReDicT) project has applied predictive analytics to help diagnose depression and identify the most effective treatment, with the overall goal of producing a commercially available emotional test battery for use in clinical settings [22]. In general, the use of ML to aggregate large datasets could significantly accelerate the diagnostic processes [23]. In Table 1, we have summarized information on ML in medicine.

Of the numerous opportunities for the use of ML in clinical practice, medical imaging workflows are those that will be likely be most impacted in the near term. ML-driven algorithms that automatically process two- or three-dimensional image scans to recognize clinical signs (e.g., tumors or lesions) or articulate diagnoses are now available and some are progressing through regulatory steps toward the market [24]. Many of these use deep learning, a form of ML based on layered representations of variables, referred to as artificial neural networks. The latter can learn extremely complex relationships between features and labels and have been shown to exceed human abilities in performing tasks such as classification of images.

ML can improve diagnostic accuracy by analyzing not only medical images but also textual records. Indeed, ML allowed the identification of varicella cases in a pediatric Electronic Medical Record Database with a positive predictive value of 63.1% and a negative predictive value of 98.8% [25].

### 6.2. Predicting Prognosis

ML has been shown to achieve the same or better prognostic definition in several clinical conditions, as compared to conventional statistical methods. In particular, ML can better predict clinical deterioration in the ward [26], mortality in acute coronary syndrome [27], survival in patients with epithelial ovarian cancer [28], complications of bariatric surgery [29], and risk of metabolic syndrome [30]. On the other hand, other studies reported that ML and conventional statistical methods have similar prognostic usefulness in predicting mortality in intensive care units [31], readmission in patients hospitalized for heart failure [32], and all-cause mortality and cardiovascular events [33].

### 6.3. Drug Discovery

ML can facilitate various phases of the early stages of drug discovery, from initial screening of drug compounds to predicted success rates based on biological factors. This includes R&D technologies like next-generation sequencing. Precision medicine, which relies on the recognition of pathophysiological mechanisms and might serve the development of alternative therapeutic pathways, appears as the most innovative area. Much of this study encompasses unsupervised learning, which is in large part still limited to identifying patterns in data without predictions (the latter is still in the realm of supervised learning). Data from experimentation or manufacturing processes have the potential to aid pharmaceutical manufacturers to diminish the time required to produce drugs, leading to lowered costs and better replication. Adopting ML approaches could play a significant role in discovering new molecules or repurposing existing drugs for rare conditions or epidemics where urgency is key. With the increase in antibiotic resistance, exploiting ML techniques is already proving quite powerful in identifying new antibacterial agents in a faster and potentially inexpensive way [23]. For example, AI recently allowed the discovery of halicin, a compound structurally divergent from conventional antibiotics, acting against *Clostridium difficile* and pandrug-resistant *Acinetobacter baumannii* infections in murine models [34].

### 6.4. Personalized Treatment

Personalized medicine, which should lead to the identification of more effective treatment based on individual health data paired with predictive analytics, is closely related to better disease assessment. To meet the complexity of personalized medicine, new types of trials have been developed, such as basket, umbrella, or platform trials. The area is presently governed by supervised learning, which permits physicians, for instance, to select from further limited sets of diagnoses or estimate patient risk based on symptoms and genetic information.

Over the next decade, the increased use of micro biosensors and devices, as well as mobile apps with more sophisticated health measurement and remote monitoring capabilities, will provide an additional surge of data that can be used to help facilitate research and development, and treatment efficacy. This type of personalized treatment has significant consequences for the individual in terms of health optimization, but also for plummeting overall healthcare costs. If more patients adhere to following prescribed drug or treatment tactics, for instance, the reduction in health care charges will trickle up and back down.

Using ML in these settings depends on the collection and analysis of huge amounts of data, but with the emergence of big data comes the challenge of statistical inference from complex datasets to identify genuine patterns, while also restraining false classifications and making decisive judgments on diagnosis and treatment possibilities. Statistical bioinformatics has proven very useful in proteomic and genomic data analysis, and the adoption of ML to build predictors and classifiers has shown significant potential [23].

## 7. Discussion

ML has the potential to transform the way medicine works [35]. However, increased enthusiasm has previously not been met by a corresponding interest from healthcare providers and operators.

*Examples where ML has done well*: Gulshan et al. have applied deep learning to build an algorithm-automated detection of diabetic retinopathy and diabetic macular edema in retinal fundus photographs [36]. Bejnordi et al. have recently evaluated the performance of automated deep learning algorithms at identifying metastases in hematoxylin and eosin-stained tissue sections of lymph nodes of women with breast cancer and related it with pathologists’ diagnoses in a diagnostic setting [37]. There are several similar ML studies on images and challenges in radiology, pathology, dermatology, ophthalmology, gastroenterology, cardiology, etc. ML is beginning to have an impact in medicine at three levels: for clinicians, predominantly via rapid, accurate image interpretation; for patients, by enabling them to process their own data to promote health; and for health systems, by improving workflow and the potential for reducing medical errors [38]. Steele et al. observed that data-driven models used on a prolonged dataset can outperform conventional models for prognosis, deprived of data pre-processing or imputing missing values for predicting patient mortality in coronary artery disease [39].

*Examples where ML has done poorly*: Esteva et al. recently demonstrated the effectiveness of deep learning in dermatology, as regards both general skin conditions and specific cancers [40]. However, they also observed that in the set of biopsy images, if an image had a ruler in it, the algorithm was more likely to call it tumor malignant because the presence of a ruler was associated with an augmented likelihood that a lesion was cancerous.

There is no clear line between ML models and traditional statistical models, and a recent article summarizes the relationship between the two [41]. However, sophisticated new ML models (e.g., those used in “deep learning” [42,43]) are well suited to learn from the complex and heterogeneous kinds of data that are generated from current clinical care, such as medical notes entered by doctors, medical images, continuous monitoring data from sensors, and genomic data to aid make therapeutically significant predictions. Most ML classifiers perform uncertainly with risk prediction. Possibly much bigger sample sizes are required to gain reliable (calibrated) risk predictions [44] than reliable (diagnostic) classifications.

ML is creating a paradigm shift in medicine, from basic research to clinical applications, but it should be carefully implemented. Vulnerabilities such as security of data and adversarial attacks, where malicious manipulation in the input can affect a complete misdiagnosis, which could be employed for fraudulent interests, present a real threat to the technology [23]. However, these vulnerabilities can be met with adequate efforts.

In the 1970s and 1980s, computerized tomography, based on the automatic elaboration of a huge bulk of X-rays images, revolutionized radio diagnostics, enabling radiologists to overcome the so-called “grey barrier”. The use of CT allowed radiologists to improve their role in the healthcare system. However, the ML revolution seems to threaten one of physicians’ most exclusive tasks, i.e., diagnostic activity. The new generation of practitioners should accept the challenge of ML, by learning how to comprehend, develop, and eventually, control it so as to improve patient care [24].

ML can analyze large amounts of data and turn that information into functional tools that can assist both doctors and patients. The increased integration of ML into everyday medical applications might improve the efficiency of treatments and lower costs in various ways. The challenge is to combine big data provided by genomics, transcriptomics, proteomics, and metabolomics with complex systems science, systems biology, and systems medicine of the body [45]. ML tools can be built for system-level interventions, comprising improving patient selection and enrolment for clinical trials, decreasing patient readmission, and automated follow-up of patients for scrutiny of complications.

## 8. Conclusions

As technology is widening and innovations and ideas are pouring, there is an enormous volume of data that is being generated in modern healthcare. Proper analytical methods are key to obtain the maximum insight from collected data.

The periphery between traditional statistics and ML is a topic to debate [46]. Some approaches fall squarely into one or the other domain, but numerous are used in both. Both statistics and ML can be of value, traditional statistics being more useful in public health and ML in omics science.

Conventional statistical approaches and ML are complementary in directing us to biologically significant conclusions: the ideal approach would be to integrate the two technologies in a way that can determine an added value. Our review has provided compelling insights into the difference between conventional statistical approaches and ML in healthcare, which in turn may help us to better integrate technology and medical care.

## Figures and Tables

**Table 1:** Applications of machine learning (ML) in medicine.

Application	Areas
Diagnostic testing	Personalized diagnostics Parkinson’s disease progression prediction from mobile phone accelerometer data Predict viral failure in AIDS patients
Medical imaging	Clinical research: MRI and PET scans and deep learning Cellular image analysis: genotype, phenotype, classification, identification, cellular tracking
Oncology	Clinical research: Identify which genes are associated with breast cancer relapse. Prognosis: Predict probability of survival in 5 years
Remote patient monitoring	Real-time predictions using data from wearables Medication adherence monitoring
